# *AHRR* Hypomethylation mediates the association between maternal smoking and metabolic profiles in children

**DOI:** 10.1097/HC9.0000000000000243

**Published:** 2023-09-27

**Authors:** Adriana C. Vidal, Shivram A. Chandramouli, Joddy Marchesoni, Nia Brown, Yukun Liu, Susan K. Murphy, Rachel Maguire, Yaxu Wang, Manal F. Abdelmalek, Alisha M. Mavis, Mustafa R. Bashir, Dereje Jima, David A. Skaar, Cathrine Hoyo, Cynthia A. Moylan

**Affiliations:** 1 Department of Biological Sciences, Center for Human Health and the Environment, North Carolina State University, Raleigh, North Carolina, USA; 2 Department of Medicine, Duke University Medical Center, Durham, North Carolina, USA; 3 Department of Obstetrics and Gynecology, Division of Reproductive Sciences, Duke University Medical Center, Durham, North Carolina, USA; 4 Division of Gastroenterology and Hepatology, Mayo Clinic, Rochester, Minnesota, USA; 5 Department of Pediatrics, Division of Gastroenterology, Hepatology, and Nutrition, Duke University Medical Center, Durham, North Carolina, USA; 6 Department of Radiology, Duke University Medical Center, Durham, North Carolina, USA; 7 Bioinformatics Research Center, North Carolina State University, Raleigh, North Carolina, USA

## Abstract

**Background:**

Tobacco smoking during pregnancy is associated with metabolic dysfunction in children, but mechanistic insights remain limited. Hypomethylation of cg05575921 in the aryl hydrocarbon receptor repressor (*AHRR*) gene is associated with *in utero* tobacco smoke exposure. In this study, we evaluated whether *AHRR* hypomethylation mediates the association between maternal smoking and metabolic dysfunction in children.

**Methods:**

We assessed metabolic dysfunction using liver fat content (LFC), serum, and clinical data in children aged 7–12 years (n=78) followed since birth. Maternal smoking was self-reported at 12 weeks gestation. Methylation was measured by means of pyrosequencing at 3 sequential CpG sites, including cg05575921, at birth and at ages 7–12. Regression models were used to evaluate whether *AHRR* methylation mediated the association between maternal smoking and child metabolic dysfunction.

**Results:**

Average *AHRR* methylation at birth was significantly higher among children of nonsmoking mothers compared with children of mothers who smoked (69.8% ± 4.4% vs. 63.5% ± 5.5, *p*=0.0006). *AHRR* hypomethylation at birth was associated with higher liver fat content (*p*=0.01), triglycerides (*p*=0.01), and alanine aminotransferase levels (*p*=0.03), and lower HDL cholesterol (*p*=0.01) in childhood. *AHRR* hypomethylation significantly mediated associations between maternal smoking and liver fat content (indirect effect=0.213, *p*=0.018), triglycerides (indirect effect=0.297, *p*=0.044), and HDL cholesterol (indirect effect = -0.413, *p*=0.007). *AHRR* methylation in childhood (n=78) was no longer significantly associated with prenatal smoke exposure or child metabolic parameters (*p*>0.05).

**Conclusions:**

*AHRR* hypomethylation significantly mediates the association between prenatal tobacco smoke exposure and features of childhood metabolic dysfunction, despite the lack of persistent hypomethylation of *AHRR* into childhood. Further studies are needed to replicate these findings and to explore their causal and long-term significance.

## Graphical abstract

**Figure:**
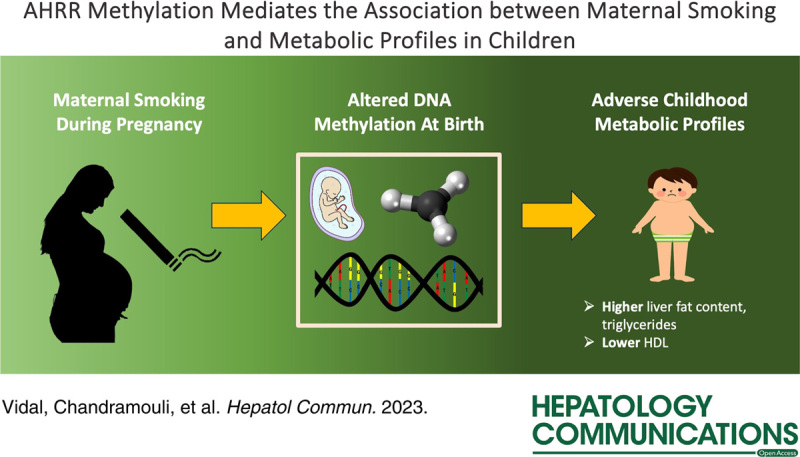

## INTRODUCTION

Maternal tobacco smoking during pregnancy remains a widely recognized public health concern.^1^ In 2020, 5.5% of the women who gave birth in the United States reported smoking during pregnancy.^2^ Prevalence of maternal tobacco smoking varies considerably based on geographic location, with rates above 15% in several states, including Missouri, Kentucky, and West Virginia.^3^

Multiple adverse health outcomes for the fetus have been associated with maternal tobacco smoking, including increased risk of preterm birth and fetal growth restriction.^4,5^ Tobacco exposure *in utero* has also been associated with adverse health effects during childhood, including respiratory symptoms such as wheezing,^6^ neurodevelopmental diseases such as attention deficit hyperactivity disorder,^7^ and substance abuse.^8,9^ Data generated in the last decade revealed significant associations between maternal smoking and child metabolic parameters at 7–12 years of age, including accelerated or ‘catch-up’ growth, an established risk factor for childhood obesity.^10,11^ More recently, we showed that prenatal tobacco smoking was associated with higher triglycerides, alanine aminotransferase (ALT) levels, and liver fat content in children. These associations varied by race, with non-Hispanic Black children having elevated ALT levels while Caucasian children had higher liver fat content.^12^ Despite mounting evidence supporting an association between *in utero* tobacco smoking exposure and childhood metabolic dysfunction, the mechanistic insights linking these remain limited.

Epigenetic alterations, such as DNA methylation, represent a possible mechanism linking maternal smoking to adverse outcomes at birth and in childhood.^13^ In a meta-analysis of 13 studies of newborns and adults,^14^ DNA methylation levels in the aryl hydrocarbon receptor repressor (*AHRR*) gene have been consistently associated with prenatal tobacco smoke exposure at several loci.^15,16^ *AHRR* is a repressor of the aryl hydrocarbon receptor (AHR), a gene linked to cholesterol homeostasis,^17,18^ hepatic steatosis, and obesity^19^ in mice and children.^20^ A bidirectional communication axis between AHR and the gut microbiome offers a molecular basis for the associations between AHR and these metabolic outcomes,^21^ and further studies have investigated the AHR-microbiome axis as a therapeutic target in fatty liver disease.^22,23^ It is possible that altered DNA methylation in *AHRR* and subsequent disruption of the AHR signaling pathway could provide a mechanistic link between metabolic dysregulation and prenatal tobacco smoke exposure.

In this study, we tested the hypothesis that hypomethylation at specific CpG sites of the *AHRR* gene mediates the association between exposure to maternal tobacco smoking during pregnancy and metabolic dysfunction in children.

## METHODS

### Study population

We leveraged data and specimen resources from the Newborn Epigenetics STudy (NEST). NEST is a longitudinal, pre-birth cohort designed to understand the role of environmental influences on epigenetic responses and health outcomes later in life. The NEST cohort was assembled between 2005 and 2011 and included pregnant women from prenatal clinics serving Duke University Obstetrics and Gynecology and Durham Regional Hospital Obstetrics facilities in North Carolina. A detailed description of the 2545 study participants, enrollment criteria and data collected have been reported.^24,25^ Children have been followed ~every 1–3 years, including at ages 1, 3, and 5. Between August 2016 and April 2018, when children were between the ages of 7–12 years, the oldest 181 with stored umbilical cord blood collected at birth were recontacted through telephone to participate in a study evaluating fatty liver disease using detailed protocols.^26^ Of the 181 children, 60 (33%) declined, 31 (17%) failed to show up for their study appointments and thus were not enrolled, leaving 90 (50%) who agreed to participate. Of these, 54 children were born to women who were body mass index (BMI) ≥30 kg/m^2^ before pregnancy, 34 were born to nonobese mothers (BMI < 30 kg/m^2^), and 2 mothers were missing prepregnancy BMI data. These analyses are restricted to 78 of the 90 children who had covariate data and imaging data of sufficient quality for inclusion in the present analysis. This study was approved by the Duke University and the North Carolina State University IRBs. Written informed consent was obtained for all participants, and children aged 12 or older provided additional assent for participation.

### Data collection

#### Covariate data

At ages 7–12 years, caretakers of the children completed a standardized questionnaire that provided information about their own and their child’s demographics, lifestyle characteristics, including employment, education, exercise, dietary habits, smoking history (currently smoke yes/no, and when they started smoking), medical comorbidities including a history of NAFLD and other chronic liver disease, family history, and medication use. These data were combined with data collected prenatally as part of the NEST cohort assembled, including demographics, medical history, and health characteristics obtained from all enrolled mothers. At delivery, parturition data were obtained from medical records.

#### Cardiometabolic outcomes at ages 7–12 years

At recontact, children underwent a physical exam which included vital signs, weight and height measurements, and waist circumference measurements. Using these values, sex and age-specific BMI percentiles were computed based on the Centers for Disease Control and Prevention protocols.^27,28^ Fasting venous blood samples were collected to measure liver function and measures of metabolic health, including ALT and aspartate aminotransferase (AST) levels, triglycerides (TG), and HDL and LDL levels.

#### Liver fat content and fibrosis assessments

Protocols for liver fat content (LFC) measurements by MRI-proton density fat fraction have been described.^19^ Briefly, LFC was assessed by MRI-proton density fat fraction using a 3.0 Tesla MRI system (TIMTrio, Siemens Healthineers, Erlangen, Germany). All scans were performed without sedation after a fasting period of more than 6 hours, and each exam lasted ~25 minutes. Abdominal adiposity was measured using a conventional, T1-weighted 3D gradient recalled echo sequence. The cross-sectional area of intra-abdominal fat, subcutaneous fat, and skeletal muscle were measured using manually drawn regions of interest. From these, we calculated subcutaneous to visceral fat and visceral to subcutaneous fat ratios.

### DNA methylation

#### Bisulfite pyrosequencing

Cord blood was collected at delivery, genomic DNA was purified from the buffy coat, and 500-ng aliquots were submitted to the Duke Genome Sciences Core Laboratory for processing using procedures described.^29^ Briefly, within minutes of delivery, the umbilical vein was punctured, and umbilical cord blood (UCB) samples were collected into K3 EDTA-treated vacutainer tubes and inverted gently to mix the anticoagulant with UCB. Specimens were transported to the laboratory, where they were centrifuged to isolate plasma and buffy coat, aliquoted, and stored at −80°C. DNA was subsequently extracted from the buffy coat using Puregene reagents according to the manufacturer’s protocol (Qiagen, Valencia, CA). Genomic DNA (800 ng) from umbilical cord blood and peripheral blood was modified with sodium bisulfite using the Zymo EZ DNA Methylation kit. Development and validation of the pyrosequencing assay for quantifying methylation at 3 CpG sites within *AHRR*, including GRCh37/hg19 chromosome 5 genomic coordinates 373,263 (position 3, corresponding to Illumina Infinium probe cg05575921), 373,240 (position 2), and 373,238 (position 1), have been described in detail.^30^ Briefly, we used forward primer 5’-TGG GGA TTG TTT ATT TTT GAG AG-3’ and reverse primer 5’-[biotin]AAA AAA CCC TAC CAA AAC CAC TC-3’ in a PCR reaction using the PyroMark PCR kit with ~20 ng of bisulfite modified DNA, assuming complete recovery, as a template. Thermocycling conditions were 95°C for 15 minutes; then 55 cycles of 94°C for 30 seconds, 58°C for 30 seconds, and 72°C for 30 seconds; then final extension at 72°C for 10 minutes. The amplicons were sequenced using primer 5’-TTG TTT ATT TTT GAG AGG GTA-3’. Pyrosequencing was performed on a Qiagen Pyromark Q96 MD instrument with percent methylation determined by Qiagen PyroMark CpG software version 1.0.11. Not all children enrolled had available blood samples at 2 time periods, thus, pyrosequencing was performed on 60 of the 78 children with available UCB at birth and all of the same 78 children at ages 7–12 years. There were 48 children with all covariate, imaging, and methylation data available from both time points of birth and ages 7–12 years.

### Statistical analysis

Caretakers’ and children’s characteristics were summarized by the mean and SD and median and IQR for asymmetric distributions. Differences in normally distributed data were analyzed using *t*- and *F*-tests, while nonnormally distributed data were analyzed using the Mann-Whitney *U* test. As appropriate, chi-square or Fisher exact test was used to compare proportions. All statistical tests were 2-sided and based on a significance level of 95%. Linear regression analyses were employed on all samples (60 children with *AHRR* methylation data measured at birth and 78 at ages 7–12 y) to maximize power. Sensitivity and mediation analyses were conducted on the 48 children for whom complete methylation, laboratory, imaging, and demographic data were available at birth and at ages 7–12 years. Linear regression models were built to test the associations between each *AHRR* position and overall metabolic parameters, including LFC, triglycerides, child BMI, AST, ALT, LDL cholesterol, and HDL cholesterol. Covariates included in these models were child sex, child age, prepregnancy maternal BMI, and maternal race/ethnicity. Linear regression models were also built to evaluate associations between maternal tobacco smoking during pregnancy and *AHRR* methylation using the same covariates. Mediation models were then built to determine the extent to which *AHRR* methylation mediated the association between maternal tobacco smoking during pregnancy and statistically significant metabolic parameters. Sensitivity analyses were performed to assess whether cotinine levels, secondhand smoking, and breastfeeding altered the associations in the regression models. Statistical power estimates for the 78 participants recontacted at ages 7–12 years ranged from 59% to 92% for models evaluating *AHRR* methylation mediation of the association between maternal smoking and childhood obesity. The power was somewhat diminished for the n=60 children whose umbilical cord blood (birth) samples were measured (range 59%–81%). These analyses were performed using SAS statistical software for Windows (version 9.4; SAS Institute, Inc., Cary, NC.).

## RESULTS

### Participant characteristics

Characteristics of the 78 mother-child dyads with usable imaging, methylation, and covariate data are summarized in Table 1. The multiethnic cohort included 67% non-Hispanic Black and 28% non-Hispanic White. Maternal tobacco smoking during pregnancy was reported by 23% of mothers, 63% reported having some college education, and 68% were overweight or obese based on prepregnancy BMI. At re-enrollment in this nested cohort study, children were a median age of 9.18 years (IQR 1.74), and 55% were female. Of 63 children with available breastfeeding data, 61% were breastfed for at least 3 months. We measured several parameters indicative of metabolic dysfunction (eg, serum triglyceride concentrations, liver enzyme levels, child weight, LFC, and serum TG/HDL ratio) at ages 7–12 years, and these are shown in Table 1. Mean values for TG were 50.86 mg/dL (SD: 21.75), LFC was 1.96% (SD: 2.11), LDL cholesterol was 87.18 mg/dL (SD: 19.48), HDL cholesterol was 55.60 mg/dL (SD: 13.17), AST was 27.70 U/L (SD: 5.18), and ALT was 17.78 U/L (SD: 5.80).

**TABLE 1:** Demographic and clinical characteristics of mother-child dyads

	N (%)	Mean ± SD
Maternal characteristics		
Race/Ethnicity		
Non-Hispanic White	22 (28.2)	—
Non-Hispanic Black	52 (66.7)	—
Hispanic	2 (2.6)	—
Other	2 (2.6)	—
Maternal smoking		
No	59 (76.6)	—
Yes	18 (23.4)	—
Education level		
High school or less	29 (37.2)	—
Some college or more	49 (62.8)	—
Prepregnancy BMI (kg/m^2^)		
≤25	24 (31.6)	—
25–30	6 (7.9)	—
>30	46 (60.5)	—
Child characteristics		
Baby sex at birth		
Male	35 (44.9)	—
Female	43 (55.1)	—
Race		
Non-Hispanic White	26 (33.3)	—
Non-Hispanic Black	52 (66.7)	
Breastfed 3+ mo		
No	22 (39.3)	—
Yes	34 (60.7)	—
Additional child characteristics		
Gestational age (wk)		
Preterm (<37 wk)	16 (20.5)	34.78±2.35
Normal (≥37 wk)	62 (79.5)	39.10±1.15
Baby weight (g)	76	3142.25±622.52
*AHRR* position methylation percent (%) at birth	58	68.30±5.96
Age 7–12 (y) child characteristics		
Child age at measured metabolic parameters	78	9.18±1.74^a^
Child weight (kg)	78	40.29±15.00
Triglycerides (mg/dL)	78	50.86±21.75
Liver fat content (%)	69	1.96±2.11
LDL cholesterol (mg/dL)	78	87.18±19.48
HDL cholesterol (mg/dL)	78	55.60±13.17
AST (U/L)	78	27.70±5.18
ALT (U/L)	78	17.78±5.80
*AHRR* position methylation (%) at ages 7–12	78	74.15±3.38

a Summary statistic reported as median + interquartile range due to asymmetric distribution.

Abbreviations: *AHRR*, aryl hydrocarbon receptor repressor; ALT, alanine aminotransferase; AST, aspartate aminotransferase; BMI, body mass index.

### *AHRR* methylation and maternal tobacco smoking

The average methylation value for all 3 CpG sites at the *AHRR* gene at birth was 68.4% (SD: 5.96), as shown in Table 1. Newborns of smoking mothers had a lower *AHRR* methylation percent at 63.5% (SE = 5.5%) compared with newborns of nonsmoking mothers at 69.8% (SE = 4.4), a 6.3% difference (SE = 5.5, *p* = 0.0006). *AHRR* methylation measured at ages 7–12 years was comparable in children of smoking mothers at 72.8% (SE = 4.8) versus children of nonsmoking mothers at 74.6% (SE = 2.8), *p* = 0.053. These differences remained unchanged after adjusting for age and sex of the child, prepregnancy maternal BMI, and race/ethnicity (Table 2).

**TABLE 2:** Regression analysis for maternal tobacco smoking and *AHRR* methylation sites

	*AHRR* methylation sites											
	Pos 1			Pos 2			Pos 3			Mean		
Maternal tobacco smoking	**Est**	**SE**	*p*	**Est**	**SE**	*p*	**Est**	**SE**	*p*	**Est**	**SE**	*p*
Birth (N = 60, Coeff. Estimate = 68.4, SD = 6)	−5.43	2.26	**0.02**	−5.77	1.85	**0.003**	−5.98	2.02	0.005	−5.73	1.82	**0.003**
Age 7–12 (N = 78, Coeff. Estimate = 74.1, SD = 3.4)	−2.39	1.40	0.09	−0.74	1.06	0.49	−0.03	1.07	0.98	−1.05	0.89	0.24

^a^Adjusted for child age, child sex, race, and maternal BMI.

Abbreviations: *AHRR*, aryl hydrocarbon receptor repressor; ALT, alanine aminotransferase; AST, aspartate aminotransferase; BMI, body mass index; Est, estimate; Pos, position.

We next evaluated whether the associations between maternal tobacco smoking during pregnancy and *AHRR* hypomethylation at the individual CpG sites were directionally consistent with *cis*-acting methylation of differentially methylated regions. We found that maternal tobacco smoking remained significantly associated with hypomethylation of newborns’ *AHRR* gene at all 3 methylation positions measured compared with newborns of nonsmoking mothers (position 1: β = −5.43, SE = 2.26, *p* = 0.02; position 2: β = −5.77, SE = 1.85, *p* = 0.003; position 3: β = −5.98, SE = 2.02, *p* = 0.005). Interestingly, while *AHRR* remained somewhat hypomethylated in the same children born to mothers who smoked tobacco during pregnancy again at ages 7–12 years, *AHRR* methylation levels were now more comparable to those children born to nonsmoking mothers (position 1: b = −2.39, SE = 1.40, *p* = 0.09; position 2: b = −0.74, SE = 1.06, *p* = 0.49; position 3: b = −0.03, SE = 1.07, *p* = 0.98).

### *AHRR* methylation and metabolic parameters

To determine if *AHRR* methylation status mediated the association between prenatal tobacco smoke exposure and metabolic dysfunction, we used regression models to first evaluate the association between *AHRR* methylation levels and parameters indicating likely metabolic disorders, including LFC, triglycerides, child BMI, AST, ALT, LDL cholesterol, and HDL cholesterol. We then assessed the extent to which *AHRR* methylation levels mediated the association between prenatal tobacco smoke exposure and metabolic dysfunction. As shown in Table 3, hypomethylation of the *AHRR* gene at birth was significantly associated with several metabolic parameters in childhood, including higher LFC (β = −0.07, SE = 0.03, *p* = 0.01), higher child BMI percentile (β = −1.47, SE = 0.69, *p* = 0.04), and higher triglycerides (β = -1.28, SE = 0.50, *p* = 0.01). In contrast, hypermethylation of *AHRR* was associated with higher HDL cholesterol (β = 0.90, SE = 0.31, *p* = 0.01). The associations between the individual CpG positions and metabolic parameters exhibited similar magnitude and directional consistency for LFC, triglycerides, and HDL, the latter of which was associated with hypermethylation at the 3 CpG sites of the *AHRR* gene. In addition, *AHRR* hypomethylation at position 3 was associated with higher ALT levels (β = −0.26, SE = 0.12, *p* = 0.03). When we included BMI percentile as a covariate in the model, results remained unchanged (Supplemental Table 1, http://links.lww.com/HC9/A483).

**TABLE 3:** Regression analysis for *AHRR* methylation sites and metabolic parameters

	*AHRR* methylation sites											
	Pos 1 (µ = 71, SD = 6.7)			Pos 2 (µ = 64.1, SD = 6.5)			Pos 3 (µ = 70.2, SD = 6.5)			Mean (µ = 68.4, SD = 6)		
	Est	SE	*p*	Est	SE	*p*	Est	SE	*p*	Est	SE	*p*
Birth (N = 60)												
Liver fat content (%)	−0.05	0.02	**0.03**	−0.06	0.03	**0.04**	−0.07	0.02	**0.01**	−0.07	0.03	**0.01**
Triglycerides (mg/dL)	−0.85	0.42	**0.05**	−1.33	0.49	**0.01**	−1.02	0.46	**0.03**	−1.28	0.50	**0.01**
Child BMI percentile	−0.83	0.61	0.18	−1.52	0.68	**0.03**	−1.57	0.66	**0.02**	−1.47	0.69	**0.04**
Child BMI (overweight)	−0.04	0.05	0.38	−0.08	0.05	0.15	−0.06	0.05	0.24	−0.07	0.06	0.20
AST (U/L)	−0.05	0.10	0.62	0.01	0.12	0.94	−0.07	0.11	0.54	−0.05	0.13	0.69
ALT (U/L)	−0.03	0.11	0.81	−0.12	0.13	0.39	−0.26	0.12	**0.03**	−0.16	0.13	0.24
LDL Cholesterol (mg/dL)	0.01	0.40	0.99	0.22	0.47	0.64	0.22	0.44	0.61	0.17	0.48	0.73
HDL Cholesterol (mg/dL)	0.48	0.27	0.08	1.01	0.30	**0.002**	0.80	0.29	**0.01**	0.90	0.31	**0.01**
Age 7–12 (N = 78)												
Liver fat content (%)	0.04	0.04	0.28	0.01	0.06	0.87	−0.01	0.05	0.80	0.04	0.07	0.59
Triglycerides (mg/dL)	−0.29	0.55	0.60	−0.60	0.73	0.41	−1.33	0.71	0.07	−1.14	0.85	0.18
Child BMI percentile	0.15	0.91	0.87	0.07	1.23	0.96	−2.10	1.23	0.09	0.04	0.08	0.59
Child BMI (overweight)	−0.03	0.05	0.59	−0.03	0.07	0.68	−0.09	0.08	0.24	−0.08	0.09	0.36
AST (U/L)	−0.11	0.11	0.33	0.04	0.15	0.78	0.06	0.15	0.68	−0.04	0.18	0.81
ALT (U/L)	0.00	0.15	0.99	−0.09	0.20	0.65	0.003	0.20	0.99	−0.04	0.24	0.86
LDL cholesterol (mg/dL)	0.03	0.48	0.94	−0.26	0.64	0.69	0.34	0.64	0.60	0.07	0.76	0.93
HDL cholesterol (mg/dL)	0.61	0.33	0.07	0.23	0.45	0.62	0.28	0.45	0.54	0.74	0.53	0.16

*Note:* Child BMI percentile was adjusted for child age, child sex, race, maternal BMI, and breastfeeding.

^a^Adjusted for child age, child sex, race, and maternal BMI.

Abbreviations: *AHRR*, aryl hydrocarbon receptor repressor; ALT, alanine aminotransferase; AST, aspartate aminotransferase; BMI, body mass index; Est, estimate; Pos, position.

The magnitude and direction of the association between *AHRR* methylation levels measured in peripheral blood in a cohort of the same children at ages 7–12 years and metabolic parameters were less consistent (Table 3). There were no statistically significant associations between mean *AHRR* methylation in peripheral blood and any metabolic parameters. Presumably, this was due to the lack of ongoing tobacco smoke exposure in childhood, as none of the children were identified as tobacco smokers themselves. Among individual CpG positions, hypomethylation at position 3 of the *AHRR* gene had a nonsignificant association with TG levels (β = −1.33, SE = 0.71, *p* = 0.07), while hypermethylation at position 1 of the *AHRR* gene had a nonsignificant association with HDL cholesterol (β = 0.61, SE = 0.33, *p* = 0.07). Restricting the analyses to only those participants without missing data (n = 48) or to those who were ever breastfed compared with not breastfed—a factor associated with protection from metabolic dysfunction—did not materially change the findings (Supplemental Table 2, http://links.lww.com/HC9/A483).

Our mediation analysis considered the direct and indirect effects of exposure to maternal tobacco smoking on metabolic parameters in children with (indirect) and without (direct) altered methylation of the *AHRR* gene. We found that methylation of the 3 CpG sites of the *AHRR* gene in umbilical cord blood at birth mediated the association between maternal smoking and LFC, TG, and HDL cholesterol in children between the ages of 7–12 years: LFC (Indirect effect = 0.213, *p* = 0.018), TG (Indirect effect = 0.297, *p* = 0.044), and HDL (Indirect effect = -0.413, *p* = 0.007), as seen in Table 4. However, *AHRR* methylation assessed in peripheral blood in the same children at ages between 7 and 12 years did not appear to mediate the relationship between maternal tobacco smoking exposure *in utero* and metabolic param*e*ters, although the direction of the associations remained the same.

**TABLE 4:** Mediation analysis of *AHRR* Methylation from maternal smoking on metabolic outcomes

	N	Direct Effect	DE *p*	Indirect effect	IE *p*
Mean *AHRR* methylation at birth (µ = 68.4, SD = 6)					
Liver fat content (%)	49	−0.071	0.726	0.213	**0.018**
Triglycerides (mg/dL)	57	0.267	0.464	0.297	**0.044**
Child BMI percentile	57	0.205	0.200	0.030	0.624
AST (U/L)	57	0.209	0.587	0.022	0.897
ALT (U/L)	57	0.281	0.445	0.111	0.464
LDL cholesterol (mg/dL)	57	−0.323	0.376	0.004	0.980
HDL cholesterol (mg/dL)	57	0.107	0.767	−0.413	**0.007**
Mean *AHRR* methylation at ages 7–12 (µ = 74.1, SD = 3.4)					
Liver fat content (%)	66	0.332	0.153	−0.032	0.514
Triglycerides (mg/dL)	75	0.622	0.034	0.045	0.434
Child BMI percentile	75	0.291	0.032	0.011	0.652
AST (U/L)	75	0.131	0.618	0.009	0.852
ALT (U/L)	75	0.326	0.300	0.002	0.958
LDL cholesterol (mg/dL)	75	−0.673	0.016	0.010	0.836
HDL cholesterol (mg/dL)	75	−0.402	0.184	−0.055	0.396

^a^Adjusted for child age, child sex, race, and maternal BMI.

Abbreviations: *AHRR*, aryl hydrocarbon receptor repressor; ALT, alanine aminotransferase; AST, aspartate aminotransferase; BMI, body mass index; DE, direct effect; IE, indirect effect.

## DISCUSSION

To our knowledge, this represents the first longitudinal study to explore *AHRR* methylation as it pertains to the association between prenatal tobacco smoke exposure and childhood metabolic outcomes. We first confirmed that *AHRR* hypomethylation in UCB at birth was associated with tobacco smoke exposure, consistent with prior studies.^15–17,31,32^ Next, we found that *AHRR* methylation in UCB was associated with multiple markers of metabolic dysfunction in childhood. Specifically, *AHRR* hypomethylation was associated with elevated LFC, triglycerides, and ALT, while *AHRR* hypermethylation was associated with elevated HDL levels. Mediation analysis then revealed that *AHRR* hypomethylation at birth significantly mediated the association between *in utero* tobacco smoke exposure and metabolic parameters in childhood.

While we could only assess association given the nature of our study, prior investigations have explored potential mechanisms that could provide a basis for these findings. One such mechanism is the bidirectional AHR-microbiome axis.^21–23^ The CpG sites identified in this study, cg05575921 and 2 adjacent sites (chr5:373,240 and chr5:373,238), regulate the expression level of *AHRR*, which in turn regulates AHR.^17^ AHR signaling has then been linked to cholesterol regulation, hepatic steatosis, and obesity in animal studies.^18–20^ In addition, *AHRR* methylation has previously been shown to mediate the relationship between maternal tobacco smoke exposure and low offspring birth weight,^33^ and children born with low birth weight then have an increased risk of childhood obesity.^9,11^ This may, in part, be explained by subclinical atherosclerosis and increased expression of inflammatory markers associated with *AHRR* methylation.^17,34^ Taken together, these findings provide a basis for exploring the utility of *AHRR* methylation monitoring in children as a tobacco smoke-related marker of metabolic disease.

While *AHRR* methylation at birth was associated with the metabolic parameters described above, *AHRR* methylation in childhood no longer carried the same associations, presumably recovering to nearly normal levels for the studied CpG sites. Prior genome-wide association studies have shown that cessation of exposure to tobacco smoke has been shown to restore methylation to nearly normal levels.^12,32,34–36^ Interestingly, despite the transient nature of the *AHRR* methylation changes present at birth in association with maternal tobacco use, these methylation marks at birth mediate the association between maternal tobacco use during pregnancy and metabolic parameters in children years later. This suggests the phenotypic impact of early epigenetic changes and their persistence later in life, even in the absence of the epigenetic changes themselves persisting.

The methylation differences described in this study can be contextualized at the molecular level. *AHRR* is located on chromosome 5, and cells are diploid at this locus. A given cell may be methylated (100% methylated), unmethylated (0% methylated), or partially methylated (50% methylated) at CpG site cg05575921, which is positioned near a tobacco smoke-inducible enhancer ^31^ that regulates *AHRR* expression and subsequently serves as negative feedback on AHR signaling. The total of *AHRR* methylation across cells analyzed in children of nonsmoking mothers was 6.3% lower than in children of smoking mothers. Studies have found that a 10% increase in methylation is associated with roughly halved *AHRR* expression in monocyte populations.^17^ Similarly, a 7.3% difference in *AHRR* methylation in umbilical cord blood was among mothers who smoked during pregnancy versus those that did not and was statistically significantly different. The clinical significance of these changes is more challenging to extrapolate and will be important to continue to assess longitudinally and in independent validation cohorts.

This study’s strength lies in its longitudinal design following the same children from prenatal tobacco smoke exposure to metabolic outcomes at ages 7–12 years. Our multifactorial assessment of metabolic outcomes, including highly accurate MRI-proton density fat fraction to quantify liver fat in addition to peripheral blood samples and BMI, further supports its measured outcomes. Limitations of the study include the relatively small sample size and the inability to make casual associations. A participation rate of ~50%, while low, is consistent with other longitudinal birth cohort studies. To assess for bias, we compared those children followed in NEST who participated in this study versus those who did not and we did not find significant demographic differences.^26^ An additional limitation is missing information on the smoking status of mothers in the postnatal setting, and as such, the impact of continued indirect smoke exposure by means of secondhand smoke could not be analyzed. Similarly, we were limited in our ability to evaluate the impact of other lifestyle factors, such as exercise and diet, given our small sample size and limited ability to control for other potential confounders. Our cohort had a high percent of non-Hispanic Black children, which is unique among pediatric fatty liver studies that typically include high numbers of Caucasian and/or Hispanic children. We were limited by sample size in our ability to assess associations within racial/ethnic groups, and we acknowledge that further studies in diverse populations are necessary.

In conclusion, this study describes a longitudinal exploration of the metabolic associations of *in utero* tobacco exposure in mother-child dyads from the prenatal period to 7–12 years of age. We found that the loss of methylation at the *AHRR* gene at birth, but not with *AHRR* methylation in childhood, mediated the association between maternal tobacco smoking during pregnancy and adverse childhood metabolic outcomes. We believe these findings demonstrate a key mechanistic underpinning for epigenetic changes that contribute to persistent metabolic impacts later in life. Larger studies with longer follow-ups are needed to confirm these findings and further explore the utility of *AHRR* methylation status as a biomarker for tobacco smoke exposure and in predicting adverse health outcomes later.

## Supplementary Material

## References

[1] Lange S Probst C Rehm J Popova S . National, regional, and global prevalence of smoking during pregnancy in the general population: A systematic review and meta-analysis. Lancet Glob Health. 2018;6:e769–e776. 29859815 10.1016/S2214-109X(18)30223-7

[2] America’s Health Rankings. Smoking During Pregnancy. United Health Foundation, 2022.

[3] Drake P Driscoll AK Mathews TJ . Cigarette Smoking During Pregnancy: United States, 2016. NCHS Data Brief. 2018:1–8. 29528282

[4] Office on Smoking and Health NCCDPHP. The Health Consequences of Smoking—50 Years of Progress: A Report of the Surgeon General. Atlanta (GA): Centers for Disease Control and Prevention (US), 2014. 24455788

[5] Office on Smoking and Health US. Women and Smoking: A Report of the Surgeon General. Atlanta (GA): Centers for Disease Control and Prevention (US), 2001. 20669521

[6] Gilliland FD Li YF Peters JM . Effects of maternal smoking during pregnancy and environmental tobacco smoke on asthma and wheezing in children. Am J Respir Crit Care Med. 2001;163:429–436. 11179118 10.1164/ajrccm.163.2.2006009

[7] Gustavson K Ystrom E Stoltenberg C Susser E Surén P Magnus P . Smoking in Pregnancy and Child ADHD. Pediatrics. 2017;139. 10.1542/peds.2016-2509 PMC5260151 28138005

[8] Rauschert S Melton PE Burdge G Craig JM Godfrey KM Holbrook JD . Maternal smoking during pregnancy induces persistent epigenetic changes into adolescence, independent of postnatal smoke exposure and is associated with cardiometabolic risk. Front Genet. 2019;10:770. 31616461 10.3389/fgene.2019.00770 PMC6764289

[9] Berlin I Oncken C . Maternal smoking during pregnancy and negative health outcomes in the offspring. Nicotine Tob Res. 2018;20:663–664. 29471548 10.1093/ntr/nty035

[10] Barker DJ . The developmental origins of well-being. Philos Trans R Soc Lond B Biol Sci. 2004;359:1359–1366. 15347527 10.1098/rstb.2004.1518 PMC1693427

[11] Ong KK . Catch-up growth in small for gestational age babies: Good or bad? Curr Opin Endocrinol Diabetes Obes. 2007;14:30–34. 17940416 10.1097/MED.0b013e328013da6c

[12] Ismaiel A Dumitrascu DL . Genetic predisposition in metabolic-dysfunction-associated fatty liver disease and cardiovascular outcomes-Systematic review. Eur J Clin Invest. 2020;50:e13331. 32589269 10.1111/eci.13331

[13] Nakamura A François O Lepeule J . Epigenetic alterations of maternal tobacco smoking during pregnancy: A narrative review. Int J Environ Res Public Health. 2021;18. 10.3390/ijerph18105083 PMC8151244 34064931

[14] Reese SE Zhao S Wu MC Joubert BR Parr CL Håberg SE . DNA methylation score as a biomarker in newborns for sustained maternal smoking during pregnancy. Environ Health Perspect. 2017;125:760–766. 27323799 10.1289/EHP333 PMC5381987

[15] Joubert BR Håberg SE Nilsen RM Wang X Vollset SE Murphy SK . 450K epigenome-wide scan identifies differential DNA methylation in newborns related to maternal smoking during pregnancy. Environ Health Perspect. 2012;120:1425–1431. 22851337 10.1289/ehp.1205412 PMC3491949

[16] Bergens MA Pittman GS Thompson IJB Campbell MR Wang X Hoyo C . Smoking-associated AHRR demethylation in cord blood DNA: Impact of CD235a+ nucleated red blood cells. Clin Epigenetics. 2019;11:87. 31182156 10.1186/s13148-019-0686-1 PMC6558773

[17] Reynolds LM Wan M Ding J Taylor JR Lohman K Su D . DNA Methylation of the Aryl hydrocarbon receptor repressor associations with cigarette smoking and subclinical atherosclerosis. Circ Cardiovasc Genet. 2015;8:707–716. 26307030 10.1161/CIRCGENETICS.115.001097 PMC4618776

[18] Dornbos P Jurgelewicz A Fader KA Williams K Zacharewski TR LaPres JJ . Characterizing the role of HMG-CoA Reductase in aryl hydrocarbon receptor-mediated liver injury in C57BL/6 Mice. Sci Rep. 2019;9:15828. 31676775 10.1038/s41598-019-52001-2 PMC6825130

[19] Moyer BJ Rojas IY Kerley-Hamilton JS Nemani KV Trask HW Ringelberg CS . Obesity and fatty liver are prevented by inhibition of the aryl hydrocarbon receptor in both female and male mice. Nutr Res. 2017;44:38–50. 28821316 10.1016/j.nutres.2017.06.002 PMC5569910

[20] Shahin NN Abd-Elwahab GT Tawfiq AA Abdelgawad HM . Potential role of aryl hydrocarbon receptor signaling in childhood obesity. Biochim Biophys Acta Mol Cell Biol Lipids. 2020;1865:158714. 32302739 10.1016/j.bbalip.2020.158714

[21] Korecka A Dona A Lahiri S Tett AJ Al-Asmakh M Braniste V . Bidirectional communication between the aryl hydrocarbon receptor (AhR) and the microbiome tunes host metabolism. NPJ Biofilms Microbiomes. 2016;2:16014. 28721249 10.1038/npjbiofilms.2016.14 PMC5515264

[22] Lin YH Luck H Khan S Schneeberger PHH Tsai S Clemente-Casares X . Aryl hydrocarbon receptor agonist indigo protects against obesity-related insulin resistance through modulation of intestinal and metabolic tissue immunity. Int J Obes (Lond). 2019;43:2407–2421. 30944419 10.1038/s41366-019-0340-1 PMC6892742

[23] Wrzosek L Ciocan D Hugot C Spatz M Dupeux M Houron C . Microbiota tryptophan metabolism induces aryl hydrocarbon receptor activation and improves alcohol-induced liver injury. Gut. 2021;70:1299–1308. 33004548 10.1136/gutjnl-2020-321565

[24] Hoyo C Murtha AP Schildkraut JM Jirtle RL Demark-Wahnefried W Forman MR . Methylation variation at IGF2 differentially methylated regions and maternal folic acid use before and during pregnancy. Epigenetics. 2011;6:928–936. 21636975 10.4161/epi.6.7.16263 PMC3154433

[25] Hoyo C Murtha AP Schildkraut JM Forman MR Calingaert B Demark-Wahnefried W . Folic acid supplementation before and during pregnancy in the Newborn Epigenetics STudy (NEST). BMC Public Health. 2011;11:46. 21255390 10.1186/1471-2458-11-46 PMC3038155

[26] Moylan CA Mavis AM Jima D Maguire R Bashir M Hyun J . Alterations in DNA methylation associate with fatty liver and metabolic abnormalities in a multi-ethnic cohort of pre-teenage children. Epigenetics. 2022:1–16. 10.1080/15592294.2022.2039850 PMC9586600 35188871

[27] Barlow SE . Expert committee recommendations regarding the prevention, assessment, and treatment of child and adolescent overweight and obesity: Summary report. Pediatrics. 2007;120 Suppl 4:S164–S192. 18055651 10.1542/peds.2007-2329C

[28] Grossman DC Bibbins-Domingo K Curry SJ Barry MJ Davidson KW Doubeni CA . Screening for obesity in children and adolescents: US Preventive Services Task Force Recommendation Statement. JAMA. 2017;317:2417–2426. 28632874 10.1001/jama.2017.6803

[29] Joubert BR Håberg SE Bell DA Nilsen RM Vollset SE Midttun O . Maternal smoking and DNA methylation in newborns: In utero effect or epigenetic inheritance? Cancer Epidemiol Biomarkers Prev. 2014;23:1007–1017. 24740201 10.1158/1055-9965.EPI-13-1256 PMC4140220

[30] Crute C Liao Y Son E Grenier C Huang Z Hoyo C . Validation of differential DNA methylation in newborns exposed to tobacco smoke during gestation using bisulfite pyrosequencing. MicroPubl Biol. 2022;2022. 10.17912/micropub.biology.000509 PMC9015814 35622517

[31] Stueve TR Li WQ Shi J Marconett CN Zhang T Yang C . Epigenome-wide analysis of DNA methylation in lung tissue shows concordance with blood studies and identifies tobacco smoke-inducible enhancers. Hum Mol Genet. 2017;26:3014–3027. 28854564 10.1093/hmg/ddx188 PMC5886283

[32] Joubert BR Felix JF Yousefi P Bakulski KM Just AC Breton C . DNA methylation in newborns and maternal smoking in pregnancy: Genome-wide consortium meta-analysis. Am J Hum Genet. 2016;98:680–696. 27040690 10.1016/j.ajhg.2016.02.019 PMC4833289

[33] Witt SH Frank J Gilles M Lang M Treutlein J Streit F . Impact on birth weight of maternal smoking throughout pregnancy mediated by DNA methylation. BMC Genomics. 2018;19:290. 29695247 10.1186/s12864-018-4652-7 PMC5922319

[34] Philibert RA Beach SR Lei MK Brody GH . Changes in DNA methylation at the aryl hydrocarbon receptor repressor may be a new biomarker for smoking. Clin Epigenetics. 2013;5:19. 24120260 10.1186/1868-7083-5-19 PMC3819644

[35] Dogan MV Shields B Cutrona C Gao L Gibbons FX Simons R . The effect of smoking on DNA methylation of peripheral blood mononuclear cells from African American women. BMC Genomics. 2014;15:151. 24559495 10.1186/1471-2164-15-151 PMC3936875

[36] Zeilinger S Kühnel B Klopp N Baurecht H Kleinschmidt A Gieger C . Tobacco smoking leads to extensive genome-wide changes in DNA methylation. PLoS One. 2013;8:e63812. 23691101 10.1371/journal.pone.0063812 PMC3656907

