# Supplementary material for: AHRR Hypomethylation mediates the association between maternal smoking and metabolic profiles in children
Source: Hepatol Commun. 2023 Sep 27;7(10):e0243. doi: 10.1097/HC9.0000000000000243 (PMC10531191; doi:10.1097/HC9.0000000000000243)
Supplement: Supplementary file 1 [file hc9-7-e0243-s001.docx]

**Supplementary Table 1. Regression Analysis for *AHRR* Methylation Sites and Metabolic Parameters Including Child BMI Percentile as Covariate**

|  | Pos 1 | | Pos 2 | | Pos 3 | | Mean | |
| --- | --- | --- | --- | --- | --- | --- | --- | --- |
| Birth (N=60) | **Est** | **P-value** | **Est** | **P-value** | **Est** | **P-value** | **Est** | **P-value** |
| Liver Fat Content (%) * | -0.05 | **0.03** | -0.06 | **0.04** | -0.07 | **0.01** | -0.07 | **0.01** |
| ** | -0.04 | 0.06 | -0.04 | 0.11 | -0.06 | **0.02** | -0.06 | **0.03** |
| LDL Cholesterol (mg/dL) * | 0.01 | 0.99 | 0.22 | 0.64 | 0.22 | 0.61 | 0.17 | 0.73 |
| ** | 0.04 | 0.92 | 0.32 | 0.52 | 0.28 | 0.53 | 0.24 | 0.62 |
| HDL Cholesterol (mg/dL) * | 0.48 | 0.08 | 1.01 | **0.02** | 0.80 | **0.01** | 0.90 | **0.01** |
| ** | 0.81 | **0.01** | 0.42 | 0.12 | 0.91 | **0.04** | 0.72 | **0.01** |
|  |  |  |  |  |  |  |  |  |
| Age 7-12 (N=78) | **Est** | **P-value** | **Est** | **P-value** | **Est** | **P-value** | **Est** | **P-value** |
| Liver Fat Content (%) * | 0.04 | 0.28 | 0.01 | 0.87 | -0.01 | 0.8 | 0.04 | 0.59 |
| ** | 0.05 | 0.19 | 0.02 | 0.69 | 0.02 | 0.69 | 0.06 | 0.31 |
| LDL Cholesterol (mg/dL) * | 0.03 | 0.94 | -0.26 | 0.69 | 0.34 | 0.60 | 0.07 | 0.93 |
| ** | 0.04 | 0.94 | -0.24 | 0.71 | 0.43 | 0.52 | 0.11 | 0.89 |
| HDL Cholesterol (mg/dL) * | 0.61 | 0.07 | 0.26 | 0.62 | 0.28 | 0.54 | 0.74 | 0.16 |
| ** | 0.60 | 0.06 | 0.19 | 0.67 | 0.04 | 0.94 | 0.61 | 0.23 |
| *Adjusted for Child Age, Child Sex, Race, and Maternal BMI | | | | | | | | |
| **Adjusted for Child Age, Child Sex, Race, Maternal BMI, and child BMI percentile | | | | | | | | |

| **Supplementary Table 2: Regression Analysis for *AHRR* Methylation Sites and Metabolic Parameters Restricted to Participants Without Missing Data** | | | | | | | | | | | | |
| --- | --- | --- | --- | --- | --- | --- | --- | --- | --- | --- | --- | --- |
|  | ***AHRR* Methylation Sites** | | | | | | | | | | | |
|  | **Pos 1** | | | **Pos 2** | | | **Pos 3** | | | **Mean** | | |
| **Birth (N = 48)** | **Est** | **SE** | **P-value** | **Est** | **SE** | **P-value** | **Est** | **SE** | **P-value** | **Est** | **SE** | **P-value** |
| Liver Fat Content (%) | -0.05 | 0.02 | **0.03** | -0.06 | 0.03 | 0.05 | -0.07 | 0.03 | **0.01** | -0.07 | 0.03 | **0.01** |
| Triglycerides (mg/dL) | -1.03 | 0.46 | **0.03** | -1.42 | 0.51 | **0.01** | -1.23 | 0.51 | **0.02** | -1.51 | 0.54 | **0.01** |
| Child BMI Percentile | -0.69 | 0.56 | 0.23 | -0.95 | 0.64 | 0.14 | -0.53 | 0.63 | 0.40 | -0.89 | 0.67 | 0.19 |
| AST (U/L) | -0.07 | 0.10 | 0.48 | 0.04 | 0.11 | 0.72 | 0.00 | 0.11 | 0.98 | -0.02 | 0.12 | 0.88 |
| ALT (U/L) | 0.00 | 0.08 | 0.97 | -0.04 | 0.09 | 0.65 | -0.14 | 0.08 | 0.11 | -0.07 | 0.09 | 0.46 |
| LDL Cholesterol (mg/dL) | 0.29 | 0.45 | 0.52 | 0.49 | 0.51 | 0.34 | 0.61 | 0.49 | 0.22 | 0.56 | 0.54 | 0.30 |
| HDL Cholesterol (mg/dL) | 0.73 | 0.28 | **0.01** | 1.09 | 0.30 | **0.001** | 1.02 | 0.30 | **0.001** | 1.15 | 0.32 | **0.001** |
|  |  |  |  |  |  |  |  |  |  |  |  |  |
|  |  |  |  |  |  |  |  |  |  |  |  |  |
| **Age 7-12 (N = 48)** | **Est** | **SE** | **P-value** | **Est** | **SE** | **P-value** | **Est** | **SE** | **P-value** | **Est** | **SE** | **P-value** |
| Liver Fat Content (%) | 0.02 | 0.04 | 0.52 | -0.04 | 0.05 | 0.46 | -0.10 | 0.05 | 0.05 | -0.05 | 0.06 | 0.47 |
| Triglycerides (mg/dL) | -1.30 | 0.71 | 0.08 | -1.37 | 0.98 | 0.17 | -1.57 | 1.01 | 0.13 | -2.66 | 1.17 | **0.03** |
| Child BMI Percentile | -0.93 | 0.86 | 0.29 | -2.43 | 1.11 | **0.03** | -2.77 | 1.13 | **0.02** | -3.44 | 1.35 | **0.01** |
| AST (U/L) | -0.17 | 0.15 | 0.26 | 0.29 | 0.20 | 0.16 | -0.09 | 0.21 | 0.67 | -0.05 | 0.25 | 0.83 |
| ALT (U/L) | 0.00 | 0.12 | 0.98 | 0.12 | 0.16 | 0.44 | -0.20 | 0.16 | 0.22 | -0.03 | 0.20 | 0.86 |
| LDL Cholesterol (mg/dL) | 0.78 | 0.68 | 0.26 | 0.45 | 0.93 | 0.63 | 0.71 | 0.95 | 0.46 | 1.29 | 1.13 | 0.26 |
| HDL Cholesterol (mg/dL) | 0.97 | 0.44 | **0.03** | 0.70 | 0.61 | 0.26 | -0.25 | 0.64 | 0.70 | 1.14 | 0.75 | 0.14 |
| *Adjusted for Child Age, Child Sex, Race, and Maternal BMI  Pos = position, Est = estimate, SE = standard error, AST = aspartate aminotransferase, ALT = alanine aminotransferase, LDL = low density lipoprotein, HDL = high density lipoprotein | | | | | | | | | | | | |
